# A tutorial and tool for exploring feature similarity gradients with MRI data

**DOI:** 10.1016/j.neuroimage.2020.117140

**Published:** 2020-11-01

**Authors:** Claude J. Bajada, Lucas Q. Costa Campos, Svenja Caspers, Richard Muscat, Geoff J.M. Parker, Matthew A. Lambon Ralph, Lauren L. Cloutman, Nelson J. Trujillo-Barreto

**Affiliations:** a Department of Physiology and Biochemistry, Faculty of Medicine and Surgery, The University of Malta, Malta; b Division of Neuroscience & Experimental Psychology, School of Biological Sciences, The University of Manchester, UK; c Institute of Neuroscience and Medicine (INM-1), Research Centre Jülich, 52425, Jülich, Germany; d Institute of Complex Systems and Institute for Advanced Simulation (ICS-2/IAS-2), Research Centre Jülich, 52425, Jülich, Germany; e Institute for Anatomy I, Medical Faculty, Heinrich-Heine-University Duesseldorf, 40221, Duesseldorf, Germany; f JARA-BRAIN, Jülich-Aachen Research Alliance, 52425, Jülich, Germany; g Centre for Medical Image Computing, Department of Computer Science, and Queen Square MS Centre, Department of Neuroinflammation, UCL Institute of Neurology, University College London, UK; h Bioxydyn Limited, Manchester, UK; i MRC Cognition and Brain Sciences Unit, University of Cambridge, Cambridge, UK

**Keywords:** Gradients, Spectral clustering, Connectivity-based parcellation, Laplacian eigenmaps, Network analysis, VB Index

## Abstract

There has been an increasing interest in examining organisational principles of the cerebral cortex (and subcortical regions) using different MRI features such as structural or functional connectivity. Despite the widespread interest, introductory tutorials on the underlying technique targeted for the novice neuroimager are sparse in the literature.

Articles that investigate various “neural gradients” (for example based on region studied “cortical gradients,” “cerebellar gradients,” “hippocampal gradients” etc … or feature of interest “functional gradients,” “cytoarchitectural gradients,” “myeloarchitectural gradients” etc …) have increased in popularity. Thus, we believe that it is opportune to discuss what is generally meant by “gradient analysis”. We introduce basics concepts in graph theory, such as graphs themselves, the degree matrix, and the adjacency matrix. We discuss how one can think about gradients of feature similarity (the similarity between timeseries in fMRI, or streamline in tractography) using graph theory and we extend this to explore such gradients across the whole MRI scale; from the voxel level to the whole brain level. We proceed to introduce a measure for quantifying the level of similarity in regions of interest. We propose the term “the Vogt-Bailey index” for such quantification to pay homage to our history as a brain mapping community.

We run through the techniques on sample datasets including a brain MRI as an example of the application of the techniques on real data and we provide several appendices that expand upon details. To maximise intuition, the appendices contain a didactic example describing how one could use these techniques to solve a particularly pernicious problem that one may encounter at a wedding. Accompanying the article is a tool, available in both MATLAB and Python, that enables readers to perform the analysis described in this article on their own data.

We refer readers to the graphical abstract as an overview of the analysis pipeline presented in this work.

## Highlights

• This article provides a beginner friendly review of the main steps for creating gradient maps.
• We introduce a new index for quantifying mesoscopic gradients (the “VB Index”).
• We use historical background to highlight the need for such quantification.
• Software for computing gradient maps and the VB Index is made available.

## Graphical abstract

The two basic algorithms to compute the VB Index and the principal gradient. The algorithm on the left depicts a searchlight algorithm that identifies local borders. The algorithm on the right (yellow route) depicts the creation of a gradient map and single VB Index for the whole brain or (inclusion of orange route) multiple clusters.

**Figure:**
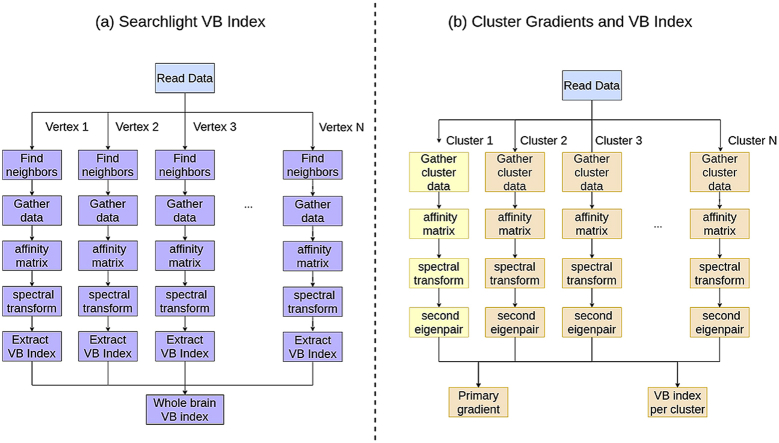

## 1 Introduction

Every discrete point in the brain (modelled as a voxel or vertex in the context of MR imaging) has several co-existing features such as the cytological architecture, the functional signature, the receptor density etc … Parcellation is one method of describing neural features and their similarities. The technique groups area of the brain that have similar features together. One of the most recognisable names in modern neuroscience is that of Korbinian Brodmann and his cytoarchitectonic parcellations of the cortex from the early 20th century. Despite his modern fame, Brodmann was not the most ardent proponent of parcellation. That honour arguably goes to Oskar and Cecile Vogt, who were the true parents of modern parcellation, and Brodmann’s mentors.

Brodmann’s regions attempted to define areas of local cytoarchitectural homogeneity in the cortex. Unfortunately, the convenience of using such areas in neuroimaging studies comes at a high price. First, Brodmann areas were defined histologically and do not necessarily correspond to gross anatomical landmarks that are visible on MRI (Zilles and Amunts, 2010). Second, Brodmann’s maps certainly are not the final word on what constitutes a cytoarchitectural brain parcel. His contemporaries (von Economo and Koskinas, 1925) as well as current researchers (Amunts et al., 2005; Bludau et al., 2014; Caspers et al., 2013, 2006; Rottschy et al., 2007; Scheperjans et al., 2008) are still investigating and refining cytoarchitectonic parcellation. Third, cytoarchitecture is not the only feature with which one can parcellate the cortex. Myeloarchitecture, for instance, has been used since the times of Brodmann (Geyer and Turner, 2013; Nieuwenhuys et al., 2015; Vogt and Vogt, 1911) and recently a vast array of neuroimaging features has been used (Glasser et al., 2016). Fourth, the boundaries (or borders) between areas characterised by any particular feature may sometimes be sharp while other times they can be blurred (Bailey and von Bonin, 1951; Brodmann, 1909; Glasser et al., 2016). Finally, different “distinct” areas may also share some relationship with other areas and thus show a non-random pattern across the whole cortex; for example functional areas involved in resting state networks (Damoiseaux et al., 2006).

In the 1950s, Percival Bailey and Gerhardt von Bonin proposed another, competing conceptualisation of cortical organisation. Vogt and Bailey can be, *prima facie,* thought of as expounding opposing points of views. While the Vogts, championed cortical parcellation, Bailey and von Bonin (1951) argue that the isocortex (or neocortex – the six layered cortex) is much more similar throughout its extent than it is different. They go so far as to state that:

“The drawing of sharp areal boundaries, on the basis of many structural peculiarities of varying distinctiveness and significance, is the fundamental defect of most maps and has been carried to absurd lengths by the Vogt school.” (p. 189)

They elaborate by stating:

“Anybody can see, to give an example, the difference between Brodmann’s areas 17 and 18. But the differences between his 18 and 19 are quite tenuous and very difficult to recognize. To draw a map on which these three areas are given three different markings - such as dots, cross-hatchings, and broken lines - is to create an entirely misleading impression. Useful as such maps are for the description of corticocortical connections, they do not translate accurately cytoarchitectonic data.” (p. viii)

An interest in similar issues of parcellations versus gradual transitions between areas has reappeared in the modern neuroscience literature. Indeed, Brodmann himself asserted that some regions demonstrated transitionary zones (Brodmann, 1909). While the feature of interest has moved on from cytoarchitecture to fMRI time series analysis or diffusion MRI tractography (more generally within a context of network analysis, connectomes and connectivity based parcellations), some themes of the early debates have lived on. For example: when, and to what extent, is clustering the cortex into distinct parcels appropriate? And, as has been explored by (Mesulam, 2008, 1998), what are the interareal relationships between cortical territories?

In 2004, a novel approach, based on spectral graph theory, appeared in the literature to investigate changes in cortical connectivity patterns across the brain using diffusion MRI tractography (Johansen-Berg et al., 2004). Similar approaches have become a popular tool for parcellating the cerebral cortex using both diffusion and functional MRI (Cloutman et al., 2012; Craddock et al., 2012; Devlin et al., 2006; Eickhoff et al., 2015; O’Donnell et al., 2013, 2006). Recently, similar techniques have also been used to explore interareal connectivity pattern changes (such as structural connectivity through tractography or functional connectivity) as one traverses the cerebral cortex; so-called feature similarity gradients (Bajada et al., 2017; Cerliani et al., 2012; Haak et al., 2018; Jackson et al., 2017; Margulies et al., 2016). Indeed, Margulies et al. (2016) have shown that, under certain constraints that likely depend on the construction of the similarity matrix, the primary rs-fMRI feature gradient reflects the interareal relationships outlined by Mesulam (2008) and elaborated by Buckner et al. (2013) where this gradient has modality selective and modality general cortices on either end. While this concept may not be fully understood by a novice reader, we hope that by the end of this article (and particularly after reading the supplementary text) the interpretation of such a statement will be obvious.

These techniques are appealing to the neuroimaging community since they have the potential to provide a flexible, unified framework for understanding similarities of neural structure or function across the brain (c.f. Paquola et al., 2020, 2019; Vázquez-Rodríguez et al., 2019 for examples of how flexible these concepts can be used across multiple modalities). In this article we further extend this framework by introducing a way to measure how sharply defined each area is, showing the full spectrum of possibilities between the ideas of the Vogts, and those of Bailey and von Bonin; The Vogt-Bailey index.

We use the historical context to highlight the importance of having a way of thinking about cortical organisation through “feature gradients” – e.g. fMRI, tractography, cytoarchtectonic etc … that bridges the gap between old debates. Specifically, we will apply the tools described here to help settle a discussion started in the middle of the 20th century. We have also made available a pair of tools written in MATLAB and Python implementing the algorithms outlined in this work, thus making it possible for the interested reader to calculate the VB index using their own data (https://doi.org/10.5281/zenodo.3609459, https://github.com/VBIndex/) (Da Costa Campos and Bajada, 2020). It is also possible to install the recommended production version of the software using Python’s *de facto* package manager, *pip*, with “*pip install vb_toolbox*”. Once this is done, the software *vb_tool* will be available for use. For usage details, we refer to the full documentation of the software’s GitHub repository (https://github.com/VBIndex/py_vb_toolbox).

We note that various groups have released their own “gradient analysis” pipelines and toolboxes including the early “gradient pipeline” by Margulies et al. (2016), connectotopic mapping focused on regional modes of connectivity changes and their statistical tests by Haak et al. (2018), LittleBrain focusing on Cerebellar gradient by Guell et al. (2019), and BrainSpace a recent all-purpose gradient toolbox by Vos de Wael et al. (2019). All these workflows and toolboxes have minor differences in certain choices that are described below. Some also include the ability to perform statistical testing on gradient maps. The software presented in this article creates similar, but not necessarily identical, ‘gradient maps’ as the other software packages available, in addition it is the only package to-date, that allows calculation of the VB index (as described in section 5.1).

In the rest of this article we describe and explore the details of the steps needed to extract feature similarity gradients and the VB Index from data. We discuss methods of measuring similarity between brain regions, why it is useful to think of these resultant similarity measurements as a mathematical graph, and how to further process the graph to obtain the desired gradient maps. In this article we restrict our discussion to a technique based heavily upon Laplacian Eigenmaps (c.f. Belkin and Niyogi, 2003, 2002). In general, the problem of finding meaningful structures and geometric descriptions of such data is usually stated as some sort of nonlinear dimensionality reduction. Although several forms of dimensionality reductions for constructing cortical gradients (or subcortical, cerebellar etc.) have been used in the literature (Coifman and Lafon, 2006; Haak et al., 2018; Johansen-Berg et al., 2004; Margulies et al., 2016), they are similar in spirit to the Laplacian Eigen-mapping reviewed here. We refer the interested reader to the relevant literature and hope that the tutorial presented here will serve as a useful introduction to understand the principles behind those related approaches.

## 2 What is a graph?

Most algorithms for feature gradient analyses emerge from the mathematical discipline of spectral graph theory. This is an approach to studying properties of graphs by computing the eigenvalues and eigenvectors of matrices that summarise the graph. While it would be lengthy to go into a detailed explanation of eigenvalues and eigenvectors in this text, we hope that their use in the context described will become clearer in later sections.

**Fig. 1:**
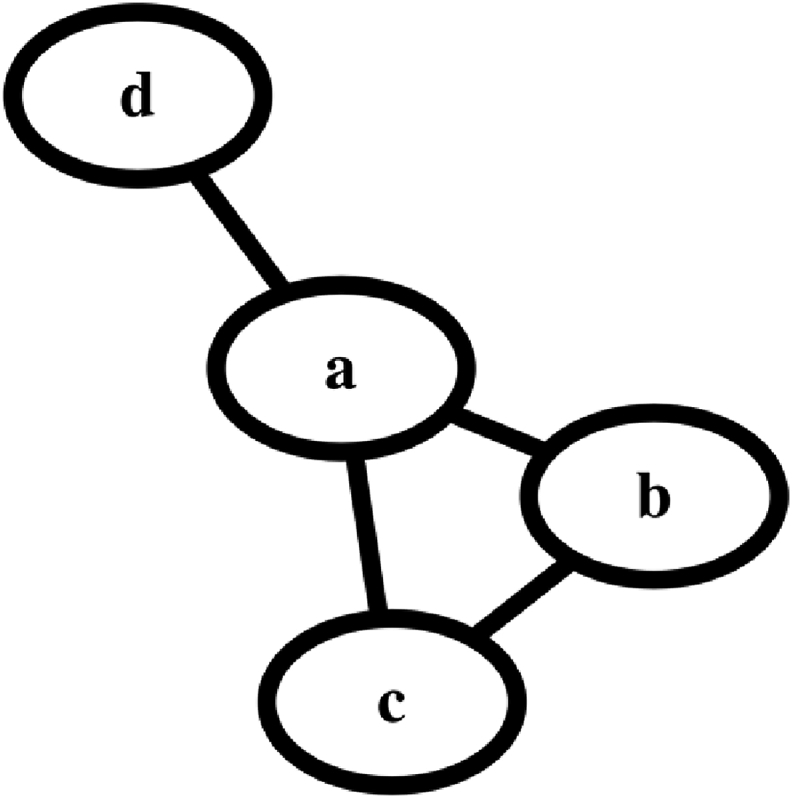
A representation of a graph with 4 nodes. Every node can be considered to be a voxel or a region of interest. The edges between the nodes represent their relationships; these can either be structural connections or a measure of similarity (affinity) between the nodes.

A graph is a mathematical structure that defines relationships between various objects. For example, the structure in Fig. 1 is a graph that defines the relationship between four objects. Each object is called a node.

The nodes could be thought of as voxels (or surface vertices) in the cortex or as cortical regions of interest.

The lines that link the nodes are called edges. The edges can be binary or have a weight associated with them (creating a weighted graph). Within neuroimaging, the edges are almost always undirected meaning that if node a connects to node b, the opposite is also true.

Some basic concepts are needed in order to proceed. The adjacency matrix is a square matrix (i.e., the same number of rows and columns) where every row and every column represent a single node, and the elements in the matrix represent the relationships between the row node and the column node. For the unweighted graph in Fig. 1, the adjacency matrix is

$$A=\left(\begin{array}{c} 0\;1\;1\;1\\ 1\;0\;1\;0\\ 1\;1\;0\;0\\ 1\;0\;0\;0 \end{array}\right).$$

The rows and columns are ordered from 1 to 4 such that entries of 1s in columns 2, 3 and 4 of row 1 means that node *a* (row 1) is connected to nodes *b, c* and *d* (columns 2, 3 and 4).

The degree matrix is a diagonal matrix where the entries along the diagonal represent the degree of each node, that is, the number of nodes that are connected (adjacent) to that node. For example, node *a* has a degree of 3 because it is adjacent to three nodes (*b, c* and *d*). The degree matrix D can be computed as the row/column wise sum of the adjacency matrix. For the graph in Fig. 1, the degree matrix is

$$D=\left(\begin{array}{c} 3\;0\;0\;0\\ 0\;2\;0\;0\;\\ 0\;0\;2\;0\\ 0\;0\;0\;1 \end{array}\right).$$

The Laplacian is defined as the degree matrix minus the adjacency matrix

L=D−A.

While the exact meaning of the Laplacian may be difficult to intuit for many readers, we hope that the use of it in Section 4 will give the readers some intuition. At this point it is useful to note that in many applications, including in neuroimaging one can define a weighted graph, where each edge connecting the nodes carry different weights. A high weight, for instance, could mean that two nodes are strongly connected, while a low weight would indicate the nodes are not as strongly connected. One can now define a weighted version of the adjacency matrix, which can be used to describe a weighted graph. In the general case, the weighted adjacency matrix can be defined as

$$W=\left(\begin{array}{cc} \begin{array}{ccc} w_{11} & w_{12} & \cdots \\ w_{21} & w_{22} & \cdots \\ \vdots & \vdots & \ddots \end{array} & \begin{array}{c} w_{1n}\\ w_{2n}\\ \vdots \end{array}\\ w_{n1}w_{n2}\cdots & w_{nn} \end{array}\right).$$

We will see later that this matrix can be associated to the concept of a similarity or affinity matrix. In the example above, the weighted adjacency matrix can be written replacing the 1s in the adjacency matrix with the corresponding weights. Weighted versions of the Degree and Laplacian matrices can be defined in the same way as before. Henceforward, unless otherwise specified, we will refer to the Laplacian, degree and adjacency matrix as their generalised weighted versions.

### 2.1 Graphs in neuroimaging

For neuroimaging purposes, a graph can be one of two distinct types. The most conceptually straightforward way of creating a graph of the brain is to consider its structural connections. For example, the nodes in the graph of Fig. 1 can represent brain areas (e.g. cerebellum, brainstem, etc …) and the edges can represent the tracts that connect those brain areas. In other words, these graphs can be obtained through tractography and assuming that each voxel (or region of interest) is a node and that every tract is an edge connecting two nodes. These graphs we refer to as *direct graphs* since the edges are the direct connections between each node. One can think of these as friendship networks where an edge between two individuals is determined on whether they are friends or not.

Another approach to constructing a graph is to compute a measure of similarity between a feature of interest of one region and the same feature of another. For structural data, the whole output of a tractography algorithm (the tractogram of a single voxel) can be considered to be a feature of a voxel and is compared pairwise to obtain a similarity matrix between voxels (Bajada et al., 2017; Cerliani et al., 2012; Devlin et al., 2006; Johansen-Berg et al., 2004). For functional data, one can use the fMRI time series. In this case, the nodes are still voxels, but the edges are weights of how similar one voxel’s feature is to another’s. If we consider that two people are nodes in a network their edge weight would be determined by how similar two individuals are, based on individual features (e.g. dress sense, job, etc …). We call this a *feature similarity graph*. For simplicity, this is the type of graph that we will be discussing in the rest of this article. It is important to note that much work in the fMRI literature performs the similarity computation not on the features themselves (e.g. the time-series), but on a “functional connectivity” matrix (c.f. Margulies et al., 2016). In terms of the “people network” proposed above, if we assume that the “functional connectivity” gives us information about the “level of friendship” between two individuals, then the similarity matrix of this last approach indicates the similarity between each individual’s friendship network.

## 3 The similarity, affinity or adjacency matrix

The adjacency matrix is a simple mathematical representation of a graph that describes the structure of the connectivity in the graph, that is, whether nodes are connected or not. A more detailed description is provided by using a weighted adjacency matrix. The question is then how to define the weights, which in turn depends on what kind of graph we want to describe.

In neuroimaging the weights can be defined in terms of a *similarity metric* describing to what extent a feature of one voxel, or vertex (e.g. an fMRI time series or a set of streamlines) is similar to every other voxel in the region of interest. This is done across all voxels (or vertices). We will refer to such a weighted adjacency matrix as the *similarity* or *affinity* matrix.

Choosing a similarity metric is extremely important since it will affect any clustering that may be done on the data. By far the most popular similarity measure between two voxels is the Pearson’s correlation coefficient, which can be interpreted as a centred and normalised dot product (Cerliani et al., 2012; Craddock et al., 2012; Devlin et al., 2006; Johansen-Berg et al., 2004; Klein et al., 2007; Zhang et al., 2014); see next section for a discussion of the dot product and other similarity measures. In order to understand the utility of correlation as a similarity metric, a short description of its precursors is given below (cf O’Connor, 2012 for an intuitive review). We will then introduce a slight modification that we employ in our adjoining code.

One caveat of the algorithms used for these analyses is that the adjacency matrix must be non-negative. This is not automatically true for most similarity measures, including those introduced in section 4.1. Hence, some technique must be used to ensure that the corresponding similarity matrix only contains non-negative weights (Haak et al., 2018; Johansen-Berg et al., 2004; Von Luxburg, 2007). For example, Johansen-Berg et al. (2004) proposed to add a scalar constant to the similarity matrix to ensure that all values are positive, others have only kept positive values at some threshold (Margulies et al., 2016).

Once a similarity matrix has been created, it can be used to represent the graph that all computations are carried out on. A final consideration regarding the similarity matrix is whether the full set of similarities should be used or if the similarity matrix should be thresholded in some way (Von Luxburg, 2007). For example, all weights below some arbitrary value ε could be set to zero; the remainder of the weights can be retained or binarised (this will be the same as using a simple adjacency matrix). Another approach to limiting the neighbourhood is to restrict the weights to the k-nearest neighbours. Advantages of both these data reduction approaches are that they remove noisy weights and they sparsify the matrix, leading to faster and cheaper computations. For example, in fMRI voxels may have a very low correlation (weight) not because of any intrinsic functional connectivity, but because of noise.

### 3.1 Similarity measures

The most basic way to measure the similarity of two datasets (thought of as vectors) is the dot product of the two vectors (consider an fMRI time series or a three-dimensional image of a tract density map (or tractogram) that is flattened into one long vector).

Geometrically, the dot product of two vectors is the projection of one vector onto the other. There are many equivalent ways to calculate the dot product. For this paper, the one offers the most insight is

$$dot\left(x,y\right)=\sqrt{\sum_{i}x_{i}^{2}}\sqrt{\sum_{i}y_{i}^{2}}\text{cos}\left(x,y\right).$$

In this form, the dot product has two components: the cosine of the angle between the two datasets (treated as vectors) and their magnitudes. This means that magnitude and angular similarity (as measured by the cosine of an angle) are confounded. In order to solve this problem, one can normalise the dot product by dividing by the magnitude of each dataset and that leaves us with the cosine function.

One problem with the cosine similarity is that it is sensitive to relative shifts in the data between samples (such as can occur in fMRI time series due to absolute signal differences that are of no interest). The most common way to create a shift invariant similarity is to mean centre the data and then compute the cosine similarity, which is the sample Pearson’s correlation coefficient:

$$corr\left(x,y\right)=\cos\left((x-\bar{x}),\left(y-\bar{y}\right)\right),$$

where $\bar{x}$ and $\bar{y}$ are constant vectors the size asx and y, where each element is the mean of x and y, respectively. Being shift invariant is an appealing property of the correlation coefficient and is especially useful to compare variables that have different means. Examples of works in the literature that use the cosine similarity can be found in the following articles (Bajada et al., 2017; Hong et al., 2019; Jackson et al., 2020, 2017; Margulies et al., 2016).

One should remember that the cosine is a sinusoidal function. As a result, a cosine similarity, or a correlation, value of 0.5 does not have the neat interpretation that the angle between the two datasets is 45°. An easy solution to this is to calculate the angle between the two data sets by using the inverse cosine function (the arccosine), normalise by 90° (or π/2):

$$normAngle=\frac{cos^{-1}\left(\cos\left((x-\bar{x}\right),\left(y-\bar{y}\right)\right))}{90}.$$

The above formula will measure a normalised “angular distance” between two datasets bound between 0 and 2. We can thus define the quantity, as follows

AngSim=1−normAngle.

This measure returns a value that has an almost identical interpretation to the correlation coefficient (or cosine similarity) but has the nice property that a value of +0.5 implies that the two datasets are half way between orthogonal and colinear while a value of −0.5 implies that the two datasets are half way between orthogonal and anti-colinear. Examples of works in the literature that use a normalised angle include (Larivière et al., 2020; Vos de Wael et al., 2018).

## 4 The spectral transformation and the graph laplacian

Once a similarity (also affinity or adjacency) matrix is computed, we have all the information that describes the relationships between individual nodes. Our next step is to embed our data into a low dimensional space (for the moment a one-dimensional line) where the nodes distances from each other and the centre of the space reflect the internodal affinity.

While we refer readers to the supplementary material (Appendix C) for an informal discussion of the problem, the process can be formulated as the solution of an optimisation problem where a suitable cost function U(x) is minimised (described by Leskovec et al., 2014)

$$\hat{x}=\underset{x}{\text{argmin}}\left\{U\left(x\right)\right\}.$$

Shortly, $\hat{x}$ is the vector that minimises the cost function U(x). Such cost function can be written as a weighted sum of squared internode (Euclidean) distances across all connected nodes,

$$U\left(x\right)=\sum_{\left(i,j\right)\in E}W_{ij}{\left(x_{i}-x_{j}\right)}^{2}.$$

One can think of the weights $W_{ij}$ as a measure of the relationship between two nodes, for example, cortical vertices. Using graph theory language, one can think of the above situation as defining a weighted graph where the value of each node represents the location of each individual and the weight of the edge connecting two nodes represent their relationship.

The minimisation of the above cost function means that long distances between pairs of vertices with a high relationship value (i.e., high weight) is penalised. As a result, a pair of vertices with a high weight will be placed close to each other, while a pair of vertices with a low weight will be placed far apart. The aim is to find a positioning where the sum of costs associated with all pairs of vertices is at its minimum.

The above problem, however, is not well-posed. First, it has a trivial solution which is to place all vertices at the same location (x is a constant vector), which produces zero cost. While this satisfies the minimisation of our cost function, it is not useful since it tells us nothing about the relationships between vertices. Second, if $\hat{x}$ is a solution then any shifting or re-scaling of $\hat{x}$by a constant value c (i.e., $\hat{y}=\hat{x}+c$ or $\hat{y}=c\hat{x}$, respectively) will also be a solution because the resulting cost function is invariant to shifting or re-scaling. Therefore, in order to circumvent trivial and non-unique solutions some constraints are required. The simplest constraint is that the cost function must be minimised subject to (s.t.) the magnitude of the position vector x being equal to 1. Mathematically this is written as

$$\hat{x}=\underset{x}{\text{argmin}}\left\{U\left(x\right)\right\}\;\;\;\;s.t.\;\;\;\;x^{T}x=1.$$

Note that this constraint does not solve the problem of a constant solution completely since a constant vector, can still produce a zero cost and also satisfy the constraint. We will see later that since this solution is known in advance, one can easily account for it after optimisation. In general, the constrained minimisation problem can be solved using the method of Lagrange multipliers (cf Hagen and Kahng, 1992). While a detailed explanation of Lagrangian multipliers is beyond the scope of this text, the modification makes the computation easier to solve while maintaining its accuracy. The idea of this method is simple: We incorporate the constraints into the cost function itself. Thus, we rewrite the problem as

$$\hat{x}=\underset{x}{\text{argmin}}\left\{U\left(x\right)+\lambda \left(1-x^{T}x\right)\right\}.$$

Now, any putative solution that does not conform to the constraint imposed will be penalised. This penalty is dictated by the weight λ, often called the Lagrange multiplier. We proceed to solve the problem in the following way. First, rewrite the optimisation problem as follows (see Appendix A for details):

$$\hat{x}=\underset{x}{\text{argmin}}\left\{\tilde{U}\left(x\right)\right\}=\underset{x}{\text{argmin}}\left\{x^{T}Lx+\lambda \left(1-x^{T}x\right)\right\}.\;$$

The minimisation can now proceed in the usual way by taking the first derivative of the modified cost function $\tilde{U}\left(x\right)$ and equating to zero (extremum condition)

$$\frac{\partial \tilde{U}\left(x\right)}{\partial x}=2Lx-2\lambda x=0,$$

and hence

Lx=λx.

The last expression defines a *standard eigenvalue problem* for the Laplacian that can be solved using standard numerical libraries (e.g., the MATLAB function *eig*). The pair $\left(\lambda ,\hat{x}\right)$ is called an eigenpair, with λ called the eigenvalue, and $\hat{x}$ the eigenvector. Eigenvalues and eigenvectors are useful in a broad range of applications, with the interpretation of these pairs depending on the context in which they are used.

In this paper, we will focus on the eigenvectors of the Laplacian, as they contain the information which we will use to create our gradients. However, the eigenvalues also encode important information. Given that the Laplacian matrix is positive semi-definite, the smallest eigenvalue is zero and its associated eigenvector is a constant, thus, we will focus upon the second smallest eigenvalue, which is termed the algebraic connectivity of a graph (Fiedler, 1973).

Fiedler (1973) showed that the magnitude of the algebraic connectivity reflects how well connected the overall graph is, i.e., the larger the algebraic connectivity is, the more difficult it is to cut a graph into independent components. If the algebraic connectivity is zero it means that the graph is not connected; i.e. there are at least two graph partitions. In other words, if a graph has at least two hard clusters (i.e. it is two completely disconnected subgraphs), the algebraic connectivity will be zero. The more connected a graph gets, the higher the algebraic connectivity becomes. This intuition will be revisited in section 5.3.

The fact that the first eigenvalue is zero directly dictates that its associated eigenvector does not carry any useful information regarding the relative position of the nodes. Hence, the optimal solution is encoded in the eigenvector associated with the second smallest eigenvalue. This is called the Fiedler vector after the mathematician who first described this solution in the context of graph partitioning (Fiedler, 1973).

At this stage, it is worth noting that the described solution to the problem is biased in the sense that nodes with high degree will dominate the minimisation since the corresponding row (or column) of the Laplacian matrix is dominant. This means that nodes with a high number of neighbours (i.e., high degree) will tend to be grouped together irrespective of their similarity. This bias can be compensated for by using a modified constraint $x^{T}Dx=1$ so that our optimisation problem is transformed to

$$\hat{x}=\underset{x}{\text{argmin}}\left\{x^{T}Lx+\lambda \left(1-x^{T}Dx\right)\right\}.$$

The new constraint means that nodes are penalised (i.e., they are assigned a higher cost) according to their degree (Johansen-Berg et al., 2004). By following the same mathematical derivation, as above, the associated eigenvalue problem is then

Lx=λDx.

This is known as the *generalised eigenvalue problem* for matrices L and D, which can also be solved numerically using standard toolboxes. Commonly, toolboxes that are able to solve the standard eigenvalue problem can also be used to solve the generalised problem. This is the case, for instance, for MATLAB’s and Scipy’s *eig* function.

Often, the Laplacian matrix is used in normalised form (i.e., normalised with respect to the nodes degree), so that its diagonal elements are all one. However, it can be demonstrated that normalising the Laplacian is equivalent to changing the constraint of the minimisation problem in some way and therefore one must be clear of how a given normalisation affects the solution. Several versions of the normalised Laplacian have been used in the literature. In Appendix B we describe the symmetric normalised Laplacian and the random walk normalised Laplacian.

## 5 Reordering, eigenmaps, and the Vogt-Bailey Index

If we think of brain voxels, or cortical surface vertices, as nodes with associated features (such as an fMRI time series, or a tractogram etc …) and the relationships between these features as edges on a graph, we previously described that the second smallest eigenvector describes the location (coordinate) of each node in one dimension, a line, which is dictated by each nodes relationship (affinity) to each-other. Using the location as a heatmap value becomes a way to visualise those relationships on the brain (the so-called macroscale gradients). Further, the components of eigenvectors denote the coordinates of the node in a space containing as many dimensions as there are eigenvectors (it is not restricted to a single dimension). Hence the eigenvector with the second smallest eigenvalue would give coordinates of the nodes on a line, the second and third eigenvectors would give the coordinates on a plane and so on. For this, more complicated visualisations are needed. One may also present the higher dimensional gradients independently, but one must always remember that the second gradient is influenced by the first and the third by the previous two etc.

Further, the algebraic connectivity indicates the sharpness of the best split (or cluster) in the region of interest. If a searchlight VB index analysis is performed on local neighbourhoods (see section 5.3), we can investigate mesoscopic gradients (or transitions between areal borders).

### 5.1 Spectral reordering

The simplest approach to mapping the embedded location onto the brain is by assigning each voxel or vertex a heatmap value that corresponds to their rank order in the embedding. This approach allows for investigating the general pattern of changes in features across the brain, but, being rank ordered, does not provide any details about the actual feature distance between vertices (c.f. Bajada et al., 2017 for an example; c.f. Johansen-Berg et al., 2004 for the original spectral reordering paper in the literature where it was used for parcellation).

### 5.2 Eigenmaps

Laplacian eigenmaps (Belkin and Niyogi, 2003, 2002) are closely related to spectral reordering. Use of eigenmaps has been introduced to the neuroscience literature (Cerliani et al., 2012; Haak et al., 2018). In one dimension, the approach uses the coordinate points given by the primary eigenvector of the Laplacian as the intensity of the voxel of interest.

This approach can be particularly advantageous since one can explore the relationships between voxels in more than one dimension. Indeed, coordinates of the similarity or eigen-space can be mapped into a colour palette and the resultant colour map value can be mapped onto the brain space by assigning that value to the corresponding voxel (c.f. Bajada et al., 2019). This means that one can only map as many dimensions as the dimensions of the colour palette (in our case the 3-dimensional RGB colour palette).

Reordering and eigenmaps give us maps of optimal embedding of voxels in a low dimensional space. Effectively, voxels (or ROIs) with a similar value, have a greater affinity to one another. This establishes the large-scale organisational gradients of the cortex. It also gives some indication as to whether there are sharp discontinuities across that gradient but a focus on the eigenvectors alone fails to quantify the extent of discontinuity in cortical intra-areal relationships. The eigenvalues provide a solution.

### 5.3 The algebraic connectivity and the Vogt-Bailey Index

The algebraic connectivity of a graph is an indicator of how “well connected” that graph is. It is the second smallest eigenvalue of the Laplacian matrix (see section 4).

Once normalised to be bounded between zero and one (by dividing by the mean of all eigenvalues save for the first, which is the maximum value a graph with an affinity matrix one ones would have), the algebraic connectivity can be used as an indicator that a particular neural region has at least one sharp delineation or comprises only graded differences. This allows for a quantification of the historical issue about the degree of interareal transitions present in the cortex. While the Vogts primarily argued for clearly demarcated brain areas, Brodmann, in his 1909 monograph clearly stated that some areal cytoarchitectonic boundaries were graded. In the extreme, Bailey and von Bonin (1951) argued for an effectively graded cortex (with some minor exceptions). We thus propose the term “Vogt-Bailey Index” to describe the normalised algebraic connectivity of the graph Laplacian when used to describe the extent of feature similarity in a neuroscientific context.

Such an approach can be done across the entire cortex to give a single value for the “gradedness” of the whole cortex, across predefined clusters (such as the resting state networks) or to give a value per region of interest that quantifies how similar features in the region are. We note, however, that this value alone tells us little since the value will be affected by smoothing (which exist in MRI signals). It can, however, be used as a relative measure where one can compare regions across the same brain or between different subjects (see section 5.3. for notes on statistical analysis).

**Fig. 2:**
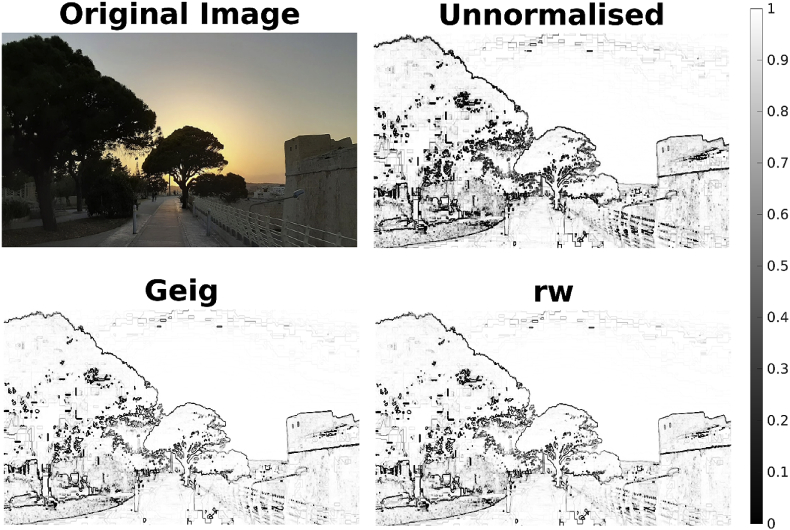
The VB Index applied to a photograph (top left) using all three normalisations of tha Laplacian matrix. The colormap ranges from black (0) where there are sharp transitions to white (1) where there is homogenous structure in the image.

**Fig. 3:**
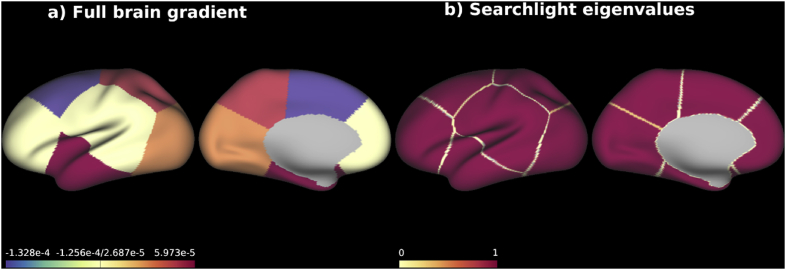
a) The gradient map on synthetic data showing the expected pattern with values within parcels being extremely similar but different across parcels. b) The VB Index on a cortical surface highlighting the arbitrary parcels. All results show the default generalised eigenvalue problem solution.

Finally, one can use a vertex-wise searchlight to calculate the Vogt-Bailey (VB) Index across the entire cortex. Using this approach, a neighbourhood of adjoining cortical voxels, or vertices (as is assumed in the adjoining code) is calculated and the (normalised) algebraic connectivity of its affinity graph is calculated. The calculated VB index gives a value of how similar a feature (fMRI, tractography, or others) in the centre of the searchlight is to its nearest neighbours. The final result is effectively a cortical edge detection algorithm (see Fig. 2, Fig. 3 for intuitive examples) where boundaries between parcels should emerge naturally and their relative sharpness should be apparent. Of course, smoothing effects and voxel/vertex size will limit the resolution that one can expect. Indeed, such notions in MRI analysis are not new. The idea of a measure of regional homogeneity (ReHo) has been present since the early days of fMRI (Jiang and Zuo, 2016; Zang et al., 2004). Further, the approach has similarities to the observer independent method for microstructural parcellations (Schleicher et al., 1999). Our approach simply fits these ideas of regional homogeneities and boundaries into a flexible and more general framework that does not restrict either the metric for similarity that is used (such as the method for microstructural parcellation) or the feature of interest (such as in both ReHo and the observationally independent approach for microstructural parcellation).

In summary, the VB Index is the proposed term for the normalised algebraic connectivity of the graph Laplacian when used to describe the extent of feature similarity in a neuroscientific context. The adjoining software can produce three “types” of VB Indices: 1) the full brain analysis which also computes a whole brain gradient single VB Index for the whole brain; 2) clustered analysis that computes a gradient and VB Index per region of interest, and 3) the searchlight VB Index which computes a VB Index per vertex based on the neighbourhood data of directly adjacent vertices. The size of the cluster (going from the nearest connected 5 or 6 neighbours to the full brain) is relevant in the interpretation of the VB Index. As Fielder (1973) showed in the original paper, the value of algebraic connectivity provides a measure of how difficult it is to split the graph (higher value indicating more “connectedness”, i.e. more difficult to split). If it is zero, then it indicates that there is at least one complete split in the graph. Hence as the cluster gets larger there is more of a chance that the graph will be easier to split into two, that is what the VB Index measures. In the case of the searchlight, since we are only looking at 5 or 6 connected neighbours, the interpretation is more straightforward: is there an edge near that vertex?

## 6 Notes on statistical analysis

This article focused on creating a conceptual understanding of the large-scale gradients and the quantification of boundary edges using MRI data.

With respect to the statistical treatment of large-scale feature gradients, a literature is emerging that discusses various statistical approaches to use on gradient maps. The reader is encouraged to explore the articles in this section for current approaches on gradient statistics (Haak et al., 2018; Hong et al., 2019; Langs et al., 2015; Tian and Zalesky, 2018; Xu et al., 2019).

Regarding the statistical analysis of the VB Index maps, future work is needed to disentangle the effects of MR noise and inherent smoothness from real gradations in feature similarity. A research avenue for noise removal includes the generation of null models with similar noise and baseline smoothness as the underlying MRI data (c.f. Gordon et al., 2016; Tian and Zalesky, 2018).

The VB toolbox is a freely available, open source, project under the terms of a GPL licence, we hope that with interest growing in the field of Gradient analysis that the toolbox will grow to also incorporate various statistical approaches for making inferences on both gradient maps and the VB Index.

## 7 Experiments

### 7.1 Photography example: searchlight VB index

Before applying the VB Index to the rather abstract notion of function MRI, we have provided a MATLAB script within the respective version of the toolbox that implements the VB Index on a colour photograph (https://github.com/VBIndex/matlab_vb_toolbox/tree/master/vb_index_intuitive_example). Every pixel within the 2D photo can be thought of as a vertex within a brain surface. The functional data is represented by the hue, saturation and their brightness value of the pixel. Performing the VB Index searchlight operation on the photograph, as described in Section 5.3. results in a quantification of boundaries of the image (See Fig. 2). Readers are invited to explore this script with other images made freely available or try it out using their own photographs.

### 7.2 A neuroimaging example: simulated MRI data example

Following the validation of the technique on colour photographs, the performance of the technique was evaluated on synthetic MRI data where the ground truth is known. To this end a cortical surface from the HCP dataset was arbitrarily split into 6 contiguous parcels. The vertices within the same parcel were assigned identical time-series, which differed across different parcels. The analysis was carried out using version 1.1.0 of the python *vb_tool.* Fig. 3 show the results of applying the proposed method to the simulated data. As expected, the full brain gradient shows a piece-wise constant pattern, reflecting the similarity structures between parcels (unknown) as described above (Fig. 3a). Consistent with this result, the vertex-wise VB Index shows a pattern where the edges between parcels are highlighted (Fig. 3b).

### 7.3 A neuroimaging example: human connectome project data example

The adjoining toolbox was run on twenty-four (24) individuals (12 ​F) from the human connectome project database. The calculations were carried out on two separate rs-fMRI runs per participant across both hemispheres. The dataset was pre-processed by the HCP using the minimal processing pipeline (Glasser et al., 2013). The data collection was approved by the Washington University Institutional Review Board (IRB) and further approval for processing the data was obtained by the University of Malta’s University Research Ethics Committee.

The data were processed according to the procedures outlined in the above text using version 1.1.0 of the python *vb_tool*; all calculations used the generalised eigenvalue problem for computations.

**Fig. 4:**
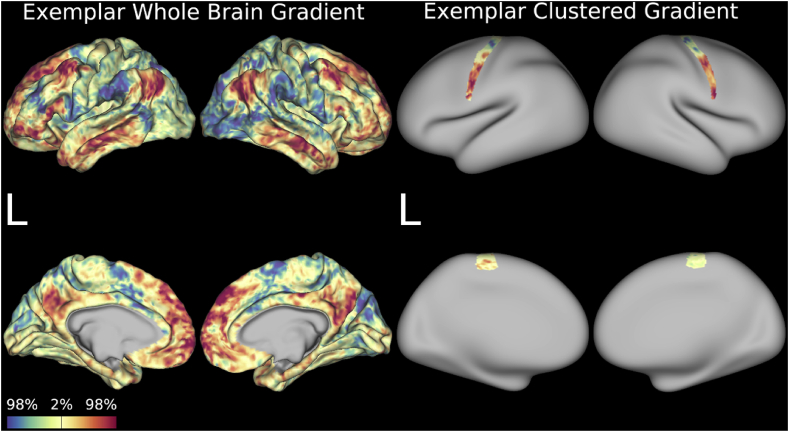
An exemplar of the principal similarity gradient across the whole cortex based on rs-fMRI as a feature (left). An exemplar of the principal gradient computed on a pre-clustered cortex (using the HCP Multimodal Parcellation, right).

**Fig. 5:**
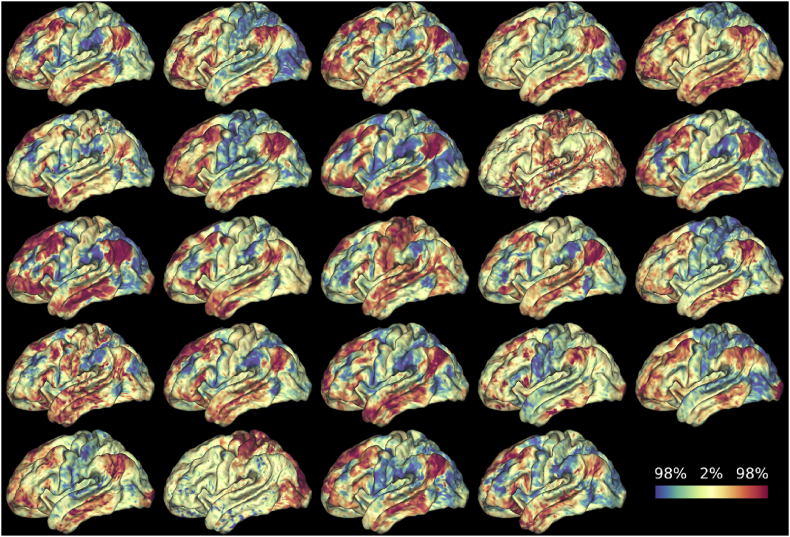
The principle similarity gradient across 24 individuals on a single run. The image of the same participants on a second fMRI run can be found in the supplementary material.

First, the whole brain affinity matrix was computed for all 24 subjects (per run) and an eigendecomposition of its Laplacian was computed. This resulted in Fig. 4 (left) which shows an exemplar of the primary large scale inter-areal (feature) gradient of the whole cortex in both hemispheres of a single subject. Fig. 5 shows all 24 subjects on the lateral surface of the left hemisphere for a single rs-fMRI run.

We note that the principle gradients differ somewhat from previously published work (c.f. Margulies et al., 2016). This may be due to multiple differences in parameter choices that were made (and discussed briefly in section 2.1). In short, the principle gradient is highly dependent upon the properties of the affinity matrix; the most pertinent difference between our affinity matrix and that of Margulies et al. (2016) was the latter’s retention of only the top 10% of functional connections and subsequent re-computation of a cosine similarity while our approach (c.f. Jackson et al., 2020, 2017 for a slightly modified example) accepts all positive correlations that were then transformed to a normalised angular distance. Thresholding plays an important role in the interpretation of the results. A high threshold (such as retaining only the top 10% of connections) will only consider the similarity of “well-connected” vertices giving no weight to moderately and poorly connected ones. Our approach, which only eliminates negative weights, takes these connections into account but would also be more sensitive to “spurious” connections.

Second, the toolbox was used to calculate the primary gradients and their associated VB Index for the data parcellated using the Multimodal HCP parcellation (Glasser et al., 2016). An exemplar of these results can be found in Fig. 3 (right), where the principle gradient is computed in each parcel. Associated with these parcels are the VB Indices per parcel (see Fig. 7 right and a further discussion below).

**Fig. 6:**
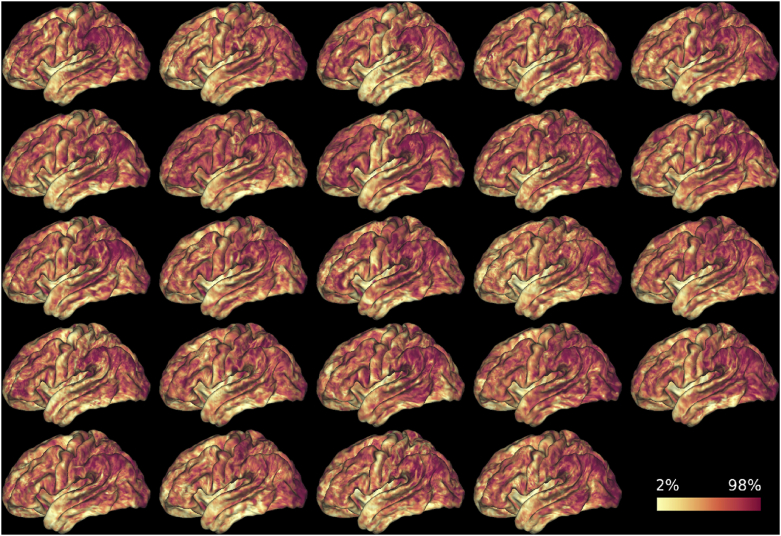
The VB Index computed across 24 individuals on a single run. The image of the same participants on a second fMRI run can be found in the supplementary material.

**Fig. 7:**
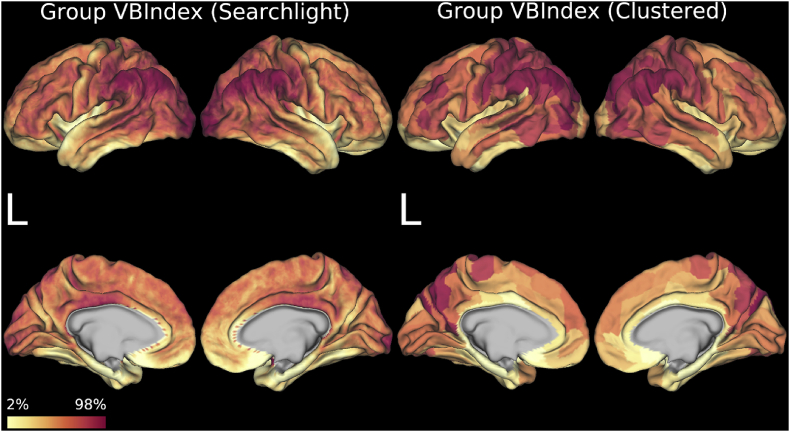
An average (on a single run) of 24 individual’s searchlight, local neighbourhood, whole brain VB-index identifying regions of relatively sharper borders across the cortex.

Finally, the vertex-wise searchlight VB Index was computed on all participants. This approach highlights, in a data-driven fashion, the feature edges and boundaries across the cortex. Fig. 6 shows the searchlight VB Index across all 24 participants while Fig. 7 (left) shows the mean vertex-wise VB Index. One can also see similar patterns between the group vertex-wise VB Index (Fig. 4 left) and the group VB Index computed on clusters (Fig. 7 right).

The full set of results can be found on the HCP BALSA database (https://balsa.wustl.edu/study/show/kND1N).

## 8 Conclusion

The idea of gradations in neural features has been present since at least the beginning of the twentieth century and has gained traction in the neuroimaging community throughout the early twenty-first. This paper has outlined the general concepts and mathematical intuition behind the spectral transformation and has introduced the related techniques of spectral reordering, Laplacian eigenmaps and clustering. As an accompaniment to this paper, MATLAB and Python tools that performs the different spectral transformations discussed in Section 4 are available. Depending on the size of the data, the technique can take up a considerable amount of RAM and computation time, however, at standard mesh sampling our attached code can run a full brain gradient analysis (using HCP 32K surfaces) on a standard desktop or notebook with 32 ​GB of RAM.

While the described framework can be used to reason about relationships between neural features, there are plenty of unanswered questions. The first important issue regards the choice of similarity measurement. Although all the metrics discussed above have been used to some extent, a systematic comparison along with guidance regarding which metric to use in different circumstances is needed.

In summary, it is hoped that this article and accompanying tools will be used as a guide to researchers interested in performing anatomical investigations using neural features and their interareal relationships in the brain.

## CRediT authorship contribution statement

**Claude J. Bajada:** Conceptualization, Data curation, Formal analysis, Investigation, Methodology, Resources, Software, Validation, Visualization, Writing - original draft, Writing - review & editing. **Lucas Q. Costa Campos:** Data curation, Formal analysis, Software, Validation, Writing - review & editing. **Svenja Caspers:** Funding acquisition, Resources, Supervision, Writing - review & editing. **Richard Muscat:** Resources, Supervision, Writing - review & editing. **Geoff J.M. Parker:** Conceptualization, Methodology, Supervision, Writing - review & editing. **Matthew A. Lambon Ralph:** Funding acquisition, Conceptualization, Supervision, Writing - review & editing. **Lauren L. Cloutman:** Funding acquisition, Conceptualization, Supervision, Writing - review & editing. **Nelson J. Trujillo-Barreto:** Formal analysis, Conceptualization, Methodology, Supervision, Writing - original draft, Writing - review & editing.

## Declaration of competing interest

GJMP is a shareholder and director of Bioxydyn, a company with an interest in imaging services and products.
