# Supplementary material for: A tutorial and tool for exploring feature similarity gradients with MRI data
Source: Neuroimage. 2020 Nov 1;221:117140. doi: 10.1016/j.neuroimage.2020.117140 (PMC7116330; doi:10.1016/j.neuroimage.2020.117140)
Supplement: Multimedia component 2 [file mmc2.docx]

**A tutorial and tool for exploring feature similarity gradients with MRI data – Supplementary Text**

Claude J. BAJADA, Lucas Q. COSTA CAMPOS, Svenja CASPERS, Richard MUSCAT, Geoff J. M. PARKER, Matthew A. LAMBON RALPH, Lauren L. CLOUTMAN, Nelson J. TRUJILLO-BARRETO

# Appendix A – The cost function

We will show that the optimisation problem

$$\hat{\boldsymbol{x}}=\underset{\boldsymbol{x}}{\mathrm{argmin}}\left\{ U\left( \boldsymbol{x} \right)+\lambda(1-\boldsymbol{x}^{T}\boldsymbol{x}) \right\}$$

can be written as an equivalent optimisation problem in terms of the graph Laplacian $L$ (as described in Leskovec et al., 2014). Where $U\left( \boldsymbol{x} \right)=\sum_{(i,j)\in E} {W_{ij}(x_{i}{-x}_{j})}^{2}.$

As stated in the text, the Laplacian is a combination of the weighted adjacency matrix and the degree matrix as follows:

$$L=D-W.$$

As can be seen, the Laplacian contains information about the degree and the adjacency of nodes. Another property of this matrix is that the rows and columns sum to zero. We can show that the original cost function $U\left( x \right)$ can be rewritten in matrix form in terms of the Laplacian (Leskovec et al., 2014) as

$$U\left( \boldsymbol{x} \right)=\boldsymbol{x}^{T}L\boldsymbol{x}.$$

This quadratic cost function can be expanded in the following way

$$U\left( \boldsymbol{x} \right){=\boldsymbol{x}}^{T}L\boldsymbol{x}=\sum_{ij}^{n} L_{ij}x_{i}x_{j}=\sum_{ij}^{n} (D_{ij}-W_{ij})x_{i}x_{j},$$

and hence

$$U\left( \boldsymbol{x} \right)=\sum_{i} D_{ii}x_{i}^{2}-\sum_{\left( i,j \right)\in E} {W_{ij}x}_{i}x_{j}.$$

Where the symbol $\left( i,j \right)\in E$ indicates that the summation runs over the edges $E$ that have a non-zero weight (i.e., the nonzero elements of the matrix $W$). Note also that the degree matrix $D$ is diagonal, and therefore the first summation runs over its diagonal elements only, so that $x_{i}{=x}_{j}$ and therefore $x_{i}x_{j}=x_{i}^{2}$. Now using the definition of the degree matrix

$$D_{kk}=\sum_{j\in E} W_{kj}=\sum_{i\in E} W_{ik},$$

where the second equality follows from the symmetry of $W$. Taking into account that multiplying the cost function by a constant does not change the solution of the minimisation problem, we have

$$U\left( \boldsymbol{x} \right)=\sum_{(i,j)\in E} W_{ij}\left( x_{i}^{2}-{2x_{i}x_{j}+x}_{j}^{2} \right).$$

Finally, one can recognise that the expression in brackets inside the summation is simply the expansion of the binomial $\left( x_{i}-x_{j} \right)^{2}$ and hence we obtain the original cost function $U\left( x \right)$

$$U\left( \boldsymbol{x} \right)=\sum_{(i,j)\in E} W_{ij}\left( x_{i}-x_{j} \right)^{2}.$$

We can then re-write the original constrained minimisation problem in terms of the Laplacian matrix (Hall, 1970) as

$$\hat{\boldsymbol{x}}=\underset{\boldsymbol{x}}{\mathrm{argmin}}\left\{ U\left( \boldsymbol{x} \right)+\lambda(1-\boldsymbol{x}^{T}\boldsymbol{x}) \right\}=\underset{x}{\mathrm{argmin}}\left\{ \boldsymbol{x}^{T}L\boldsymbol{x}+\lambda{(1-\boldsymbol{x}}^{T}\boldsymbol{x}) \right\}.$$

# Appendix B – Normalised Laplacians

## 10.1. The Random Walk Normalised Laplacian

The random walk Laplacian (Von Luxburg, 2007) is defined by pre-multiplying the Laplacian by the reciprocal of the degree matrix

$$L^{rw}=D^{-1}L.$$

It can be shown that solving the standard eigenvalue problem using the random walk normalised Laplacian is equivalent to solving the required generalised eigenvalue problem for the un-normalised Laplacian. Pre-multiplying by $D$ both sides of the standard eigenvalue problem for $L^{rw}$ we have

$$DL^{rw}\boldsymbol{x}=\lambda D\boldsymbol{x},$$

which reduces to the generalised eigenvalue problem for the un-normalised Laplacian $L$.

$$L\boldsymbol{x}=\lambda D\boldsymbol{x}.$$

This means that by solving the standard eigenvalue problem for the random walk Laplacian in effect changes the constraint of the cost function in a way that automatically accounts for the node degree bias.

## 10.2. The Symmetric Normalised Laplacian

The symmetric Laplacian is another very popular normalisation. It can be obtained by pre- and post-multiplying the Laplacian by the square root of the reciprocal of the degree matrix

$$L^{sym}=D^{-\frac{1}{2}}LD^{-\frac{1}{2}}$$

where the pre and post multiplication ensures symmetry, which is a desirable property from a numerical point of view when solving eigenvalue problems. To understand how this normalisation affects the solution, we proceed in the same way as for the random walk Laplacian and start by writing down the standard eigenvalue problem for $L^{sym}$

$$L^{sym}\boldsymbol{y}=\lambda\boldsymbol{y}.$$

We have used a different variable name $y$ to indicate that the solution to this problem may be different from the solution for the random walk Laplacian. Using the definition of $L^{sym}$ and pre-multiplying this expression by $D^{\frac{1}{2}}$ we have

$$LD^{-\frac{1}{2}}\boldsymbol{y}=\lambda D^{\frac{1}{2}}\boldsymbol{y}.$$

We can easily see now that this problem can be transformed into the generalised eigenvalue problem for the un-normalised Laplacian by using the change of variables $\boldsymbol{y}=D^{\frac{1}{2}}x$. Substituting for the new variable $\boldsymbol{x}$, we obtain

$$L\boldsymbol{x}=\lambda D\boldsymbol{x}.$$

This means that one can obtain the bias corrected solution $x$ by first solving the standard eigenvalue problem for $L^{sym}$ (i.e., obtaining the biased solution $y$ for $L^{sym}$), and then applying the inverse transformation $x=D^{-\frac{1}{2}}y$ to recover the bias corrected solution.

While each approach to normalising the Laplacian is different, the result is the same in each case. Most neuroimaging work uses some form of the normalised Laplacian and it seems that the symmetric normalisation tends to be most commonly used (Devlin et al., 2006; Johansen-Berg et al., 2004; Klein et al., 2007).

# Appendix C – An Intuitive Explanation of the Spectral Transformation: The problem of the guests at a wedding

For didactic purposes, let us imagine a scenario where Oskar and Cecile Vogt’s best friend “Fred” is marrying Percival Bailey and Gerhardt von Bonin’s student “Martha”. Martha and Fred decide to have a very small wedding and only invite the above-mentioned individuals.

Let us also assume that we are, inexplicably, given the task of seating these guests at the wedding table. We soon realise that the task is not well specified since there are many ways of organising our guests on the table (a mathematically ill-posed problem). We could group them at random, or by height, shoe size, dress sense, etc. However, to avoid the obvious frictions that could arise by sitting Cecile Vogt next to Gerhardt von Bonin, we come up with the clever and promising idea of grouping them according to some measure of “affinity”.

We will show that, by treating the guests’ positions as values of nodes in a graph, and their mutual affinity (i.e. their social network similarity) as connections’ strengths between the nodes, the wedding table problem can be stated as an equivalent optimisation problem, the solution of which will place guests with great affinity for each other close together, while guests with low affinity will be far apart.

The matrix in Figure S1 sets up the imaginary scenario threaded through this article showing the relationship values between the members of the wedding party where both the Vogts and Bailey, von Bonin duo are invited.

In this particular wedding, we will assume that the bride and groom would really want to sit next to one another (and hence have a very high affinity value) whereas the Vogts really do not want to sit next to their rivals and have a low affinity value. The affinity is never lower than .4 because they are all civilised human beings!

While the matrix in Figure S1 gives all the information regarding the relationships between people, when the number of people (or nodes) becomes large, all this information becomes very difficult to interpret. The rationale behind the spectral transformation is to embed each node in a lower dimensional coordinate system where the distance between points in this space reflects their similarity, thereby simplifying the presentation of the information.

To simplify matters, let us consider embedding the nodes into a one-dimensional coordinate system (a line, or a traditional trestle wedding table).


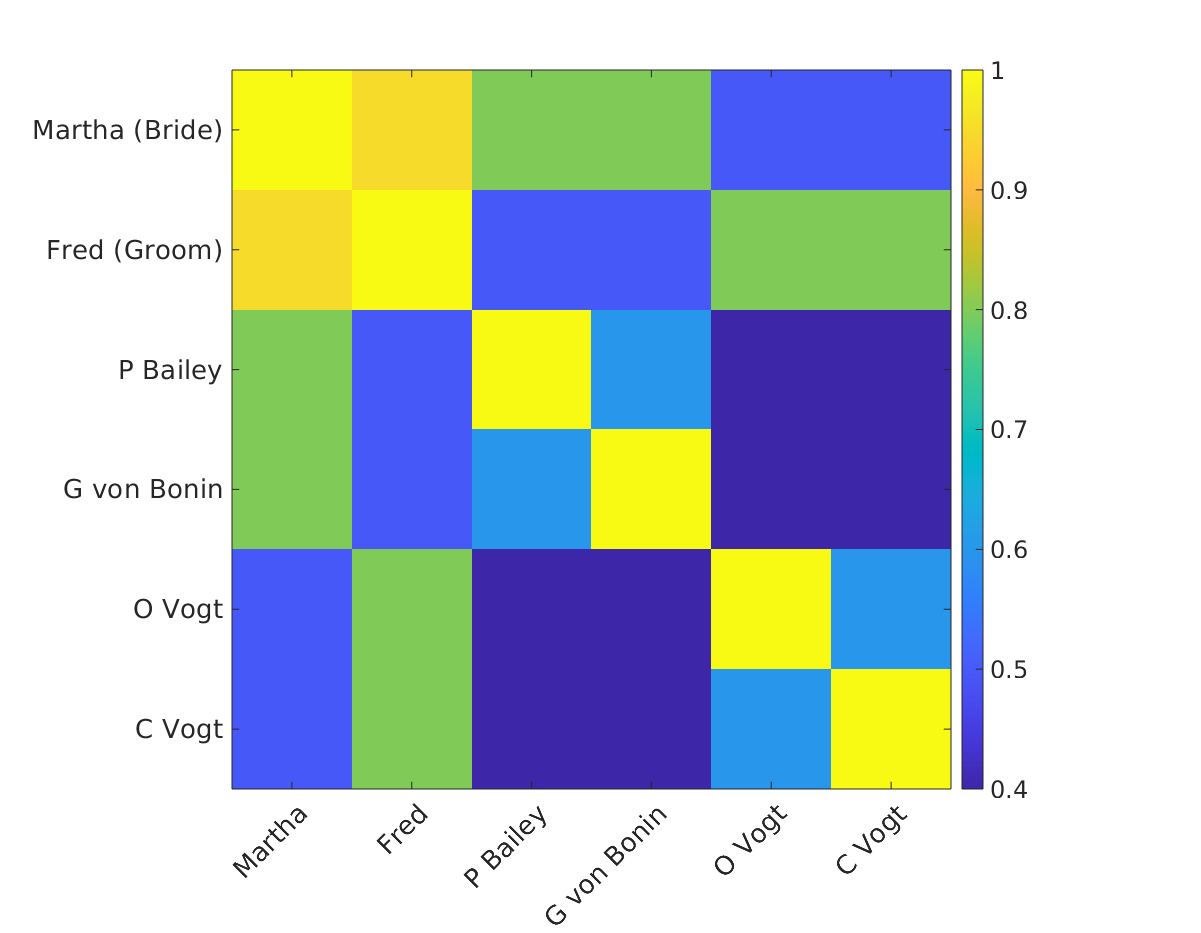


**Figure S1: Similarity matrix showing the relationships between people who are to sit on the trestle wedding table**

Imagine a long table where all these guests are to sit. Think of the centre of this table as the zero point. An optimal way to sit the people (nodes) at this table is to place them on either side of the centre such that people who have the closest relationship are close together on the table and that people on one side of the centre of the table have as little similarity to the people of the other side of the zero point as possible. One way to obtain such a layout is to place a greater penalty on positioning people with a high relationship far away from each other in the seating arrangement

$$\hat{\boldsymbol{x}}=\underset{\boldsymbol{x}}{\mathrm{argmin}}\left\{ U\left( \boldsymbol{x} \right) \right\},$$

where

$$U\left( \boldsymbol{x} \right)=\sum_{(i,j)\in E} {W_{ij}(x_{i}{-x}_{j})}^{2}.$$

In the main text, we saw that the position (or ordering) was defined by *x* (the eigenvectors) and that the optimal solution was given by the eigenvector with the second smallest eigenvalue. Furthermore, the components of eigenvectors denote the coordinates of the node in a space containing as many dimensions as there are eigenvectors. Hence the eigenvector with the second smallest eigenvalue would give coordinates of the nodes on a line, the second and third eigenvectors would give the coordinates on a plane and so on.

If one thinks of the wedding problem described earlier, the eigenvector of the matrix in Figure S1 associated with the second smallest eigenvalue will denote the position that each wedding guest will sit at (Table S1).

**Table S1: Table showing position and order of guests at the head table**

| Guest | Position | Rank of Position |
| --- | --- | --- |
| Bride | -0.1707 | 3 |
| Groom | 0.1707 | 4 |
| Bailey | -0.4852 | 1 |
| von Bonin | -0.4852 | 1 |
| O Vogt | 0.4852 | 5 |
| C Vogt | 0.4852 | 5 |

Since it is customary for each guest at the table to sit at a fixed distance from one another, the exact position is irrelevant, and a rank of position can be taken as a sufficient descriptor. In this case we can see that one can start with the von Bonin and Bailey, and then sits the bride and groom followed by the Vogts. Note that in this case each couple have interchangeable seats (since they have the same position values). Also, the order rather than the actual distance between nodes is what one is investigating. Once the optimal order has been found, the original similarity matrix can be permuted to reflect the ordering (Figure S2). We order the elements in the matrix according to the optimal one-dimensional ordering. After re-ordering, we can now easily identify two clusters. The top left quadrant of the matrix shows the grouping of the bride’s family while the bottom left shows the grouping of the groom’s.


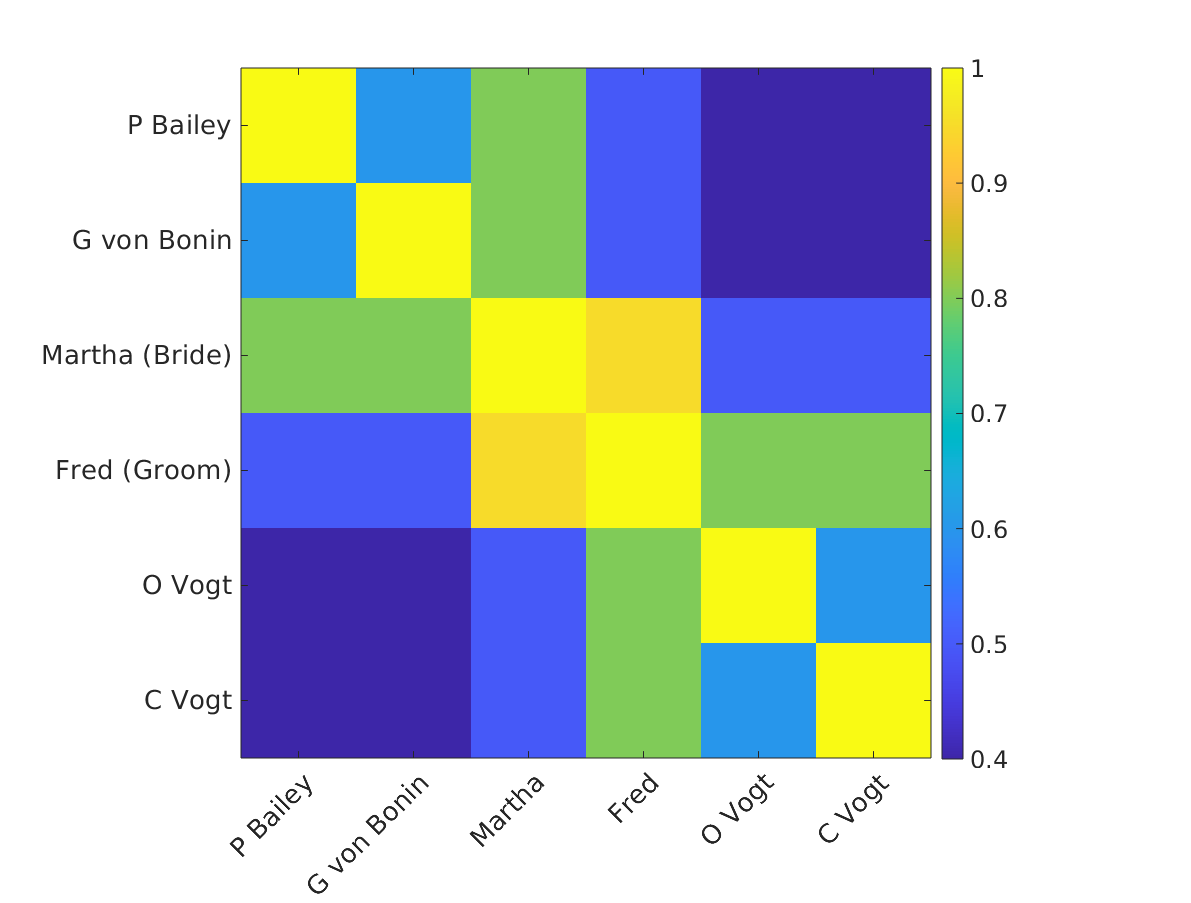


**Figure S2: reordered matrix showing groups of guests**

In the case of Laplacian Eigenmaps**,** the guests would not sit at equidistant positions but in the exact positions denoted by their associated eigenvector element. These positions are shown in column 2 of Table 1. In the a two-dimensional eigenmap is akin to the wedding party getting off the table (1 dimension), setting up for the first dance (2 dimensions) etc.

# Appendix D - Clustering

Sometimes, having clear and distinct brain regions (or parcels) is useful, even if it may not necessarily be the best representation of ground truth.

In fact, all of the common clustering algorithms such as k-means, fuzzy c-means, Gaussian mixtures and hierarchical clustering can be applied to the transformed data, where each component of the $k$ smallest eigenvectors is treated as the coordinates of a point in a Euclidean space (ℝ^k^). As we've previously stated, the spectral transformation is a way to transform the original similarity data into a new space (the eigen-space) where data points are grouping data points according to their similarity.

Let us consider clustering in the context of the wedding table analogy previously discussed. Whereas spectral reordering and Laplacian eigenmaps were primarily interested in understanding where people should sit at the table, a clustering technique could be applied after the guests are sitting at the table (i.e., after that spectral transformation), in order to put the guests into distinct groups. In our example, if the guests were to be put into two groups (or clusters), then they may be the bride’s and the groom’s friends. If three groups were considered instead, then one may separate the guests into the Vogts, Bailey and von Bonin, and the wedding couple.

# Appendix E – A note on the name Vogt-Bailey Index

We would finally like to note the difficulty in choosing a concise name for the index. Of the four people referred to, both Vogts could be represented in a single word, however, the difficulty arose in whether to call the index the Vogt-Bailey or Vogt-Bonin index. While Percival Bailey was the first author of the 1951 monograph on the Isocortex of man, Bailey was a neurosurgeon whose primary research interest was on tumours (Ferguson and Lesniak, 2005). von Bonin, was the first author on the earlier monograph on the cortex of the macaque where the idea of gradients was introduced (von Bonin and Bailey, 1947). To this end the choice of name was, admittedly, arbitrary and the authors are happy for the index to alternately be called the Vogt Bonin Index by anyone who feels that von Bonin is more deserving; as long as the abbreviation remains VB for clarity and consistency.

**Bibliography**

Devlin, J.T., Sillery, E.L., Hall, D.A., Hobden, P., Behrens, T.E.J., Nunes, R.G., Clare, S., Matthews, P.M., Moore, D.R., Johansen-Berg, H., 2006. Reliable identification of the auditory thalamus using multi-modal structural analyses. Neuroimage 30, 1112–1120. doi:10.1016/j.neuroimage.2005.11.025

Ferguson, S., Lesniak, M.S., 2005. Percival Bailey and the classification of brain tumors. Neurosurg. Focus 18, e7. doi:10.3171/foc.2005.18.4.8

Hall, K.M., 1970. An r-Dimensional Quadratic Placement Algorithm. Manage. Sci. 17, 219–229.

Johansen-Berg, H., Behrens, T.E.J., Robson, M.D., Drobnjak, I., Rushworth, M.F.S., Brady, J.M., Smith, S.M., Higham, D.J., Matthews, P.M., 2004. Changes in connectivity profiles define functionally distinct regions in human medial frontal cortex. Proc. Natl. Acad. Sci. USA 101, 13335–13340. doi:10.1073/pnas.0403743101

Klein, J.C., Behrens, T.E.J., Robson, M.D., Mackay, C.E., Higham, D.J., Johansen-Berg, H., 2007. Connectivity-based parcellation of human cortex using diffusion MRI: Establishing reproducibility, validity and observer independence in BA 44/45 and SMA/pre-SMA. Neuroimage 34, 204–211. doi:10.1016/j.neuroimage.2006.08.022

Leskovec, A., Rajaraman, A., Ullman, J.D., 2014. Mining of Massive Datasets. Cambridge University Press, Cambridge. doi:10.1017/CBO9781139058452

Von Luxburg, U., 2007. A tutorial on spectral clustering. Stat Comput 17, 395–416. doi:10.1007/s11222-007-9033-z

von Bonin, G., Bailey, P., 1947. The neocortex of Macaca mulatta, Illinois monographs in the medical sciences. Urbana : University of Illinois Press.
